# New Approaches in Detection and Treatment of Familial Hypercholesterolemia

**DOI:** 10.1007/s11886-015-0665-x

**Published:** 2015-10-19

**Authors:** Merel L. Hartgers, Kausik K. Ray, G. Kees Hovingh

**Affiliations:** Department of Vascular Medicine, Academic Medical Center, Meibergdreef 9, 1105 AZ Amsterdam, The Netherlands; Department of Primary Care and Public Health, School of Public Health, Imperial College London, London, UK

**Keywords:** Familial hypercholesterolemia, Diagnosis, Treatment, Hyperlipidemia

## Abstract

Familial hypercholesterolemia (FH) is an autosomal dominant genetic disorder that clinically leads to increased low density lipoprotein-cholesterol (LDL-C) levels. As a consequence, FH patients are at high risk for cardiovascular disease (CVD). Mutations are found in genes coding for the LDLR, apoB, and PCSK9, although FH cannot be ruled out in the absence of a mutation in one of these genes. It is pivotal to diagnose FH at an early age, since lipid lowering results in a decreased risk of cardiovascular complications especially if initiated early, but unfortunately FH is largely underdiagnosed. While a number of clinical criteria are available, identification of a pathogenic mutation in any of the three aforementioned genes is seen by many as a way to establish a definitive diagnosis of FH. It should be remembered that clinical treatment is based on LDL-C levels and not solely on presence or absence of genetic mutations as LDL-C is what drives risk. Traditionally, mutation detection has been done by means of dideoxy sequencing. However, novel molecular testing methods are gradually being introduced. These next generation sequencing-based methods are likely to be applied on broader scale once their efficacy and effect on cost are being established. Statins are the first-line therapy of choice for FH patients as they have been proven to reduce CVD risk across a range of conditions including hypercholesterolemia (though not specifically tested in FH). However, in a significant proportion of FH patients LDL-C goals are not met, despite the use of maximal statin doses and additional lipid-lowering therapies. This underlines the need for additional therapies, and inhibition of PCSK9 and CETP is among the most promising new therapeutic options. In this review, we aim to provide an overview of the latest information about the definition, diagnosis, screening, and current and novel therapies for FH.

## Introduction

Familial hypercholesterolemia (FH), a common autosomal dominant inherited disorder, is characterized by high plasma levels of low density lipoprotein-cholesterol (LDL-C) and, as a consequence, high risk for the premature development of atherosclerosis and cardiovascular disease (CVD) [1]. The pathological substrate of FH is related to the dysfunctional uptake of LDL particles via its receptor and this can either be caused by mutations in the genes encoding for the LDL receptor (LDLR), apolipoprotein B (apoB), or pro-protein convertase subtilisin/kexin 9 (PCSK9). It is important to diagnose FH at an early age in order to prevent vascular events. The diagnosis is based on clinical parameters such as lipid levels, presence of xanthomas, family history, and vascular disease, and a definite diagnosis is based either on the identification of a pathogenic mutation in any of the three well-established FH-causing genes or a probably score derived from clinical characteristics [2]. It has also been postulated that a polygenic form of FH is present in patients meeting the clinical criteria for FH (i.e., according to the Dutch Lipid Criteria Score, Simon Broome Criteria) who do not carry a mutation in one of these genes [3]. There is a wide range in the lipid levels among patients with FH, and this is largely related to the severity of the mutation and the specific gene; patients carrying a mutation in the *LDLR* gene, for example, tend to suffer from a more severe phenotype than *APOB* mutation carriers [4]. The CVD outcome differs among heterozygous carriers of FH mutations, who, in general, typically suffer from CVD events in their fourth decade of life, while patients suffering from homozygous FH, the much rarer form of FH, might already have experienced serious cardiovascular complications in the second decade of life or even in childhood [5].

HMG-coenzyme reductase inhibitors (“statins”) are the therapy of first choice in FH patients [6]. It is of note however that both the magnitude of CVD risk in untreated FH patients, as well as the CVD risk reduction of statins, is not well-established as randomized controlled trials have not been conducted in this regard. We aim to provide a comprehensive overview of the pathophysiology, epidemiology, screening programs as well as current and future therapies of FH.

## Pathophysiology and Genetics

### *LDLR*

FH is caused by a mutation in the gene encoding the LDLR in more than 90 % of the molecular diagnosed cases, and this mutation leads to absent or dysfunctional LDLR at the surface of the hepatocytes [7]. As a consequence, hepatic uptake of LDL-C is decreased which results in elevated plasma levels of LDL-C [1]. The *LDLR* gene is located on the short arm of chromosome 19, and to date, over 1700 mutations in the *LDLR* gene have been described (http://www.ucl.ac.uk/ldlr/Current/). Five different classes of *LDLR* mutations have been identified, dependent on the effect on the phenotype. Class 1 mutations are null mutations that result in no detectable LDLR protein. In class 2 mutations, the transport of the LDLR from the endoplasmic reticulum to the Golgi apparatus is blocked completely (class 2a) or partially (class 2b). A class 3 mutation leads to expression of a non-functional LDLR. Class 4 mutations result in LDL binding but the LDLR-LDL complexes cannot be internalized, and in class 5 mutations, recycling of the LDLR is not efficient and therefore do not reach the cell surface [8].

### *APOB*

In 5 % of the molecular-diagnosed FH cases, a pathogenic mutation is found in the gene encoding for apoB and this disease is also referred to as familial defective apoB [9]. The impaired binding of LDL particles to the LDLR therefore results in higher circulating LDL-C concentrations.

### *PCSK9*

In 2003, gain-of-function mutations in a third gene, encoding for PCSK9, were identified as a cause of FH [10]. PCSK9, when forming a complex with the LDLR, is internalized by modification of the LDLR confirmation and interferes with LDLR recycling. This leads to LDLR degradation and therefore reduction of the amount of receptors available at the hepatocyte surface to bind circulating LDL particles [10].

### Homozygous FH

Patients suffering from homozygous FH (HoFH) are characterized by severely elevated LDL-C levels (typically above 13 mmol/L), and due to this extreme dyslipidemia, patients have been reported to suffer cardiovascular events in the first decade of life [11]. The molecular defect can either be caused by homozygosity or, more frequently, compound heterozygosity for mutations predominantly in the *LDLR* gene. Moreover, combined mutations in *APOB* and *PCSK9* have also been described (double heterozygotes) [11].

## Epidemiology

Unfortunately, the number of individuals diagnosed with FH in most countries is <1 %, except for countries where active screening does take place such as in the Netherlands and Norway where 71 and 43 % of the patients have been described to be diagnosed [7]. These numbers, however, were based on an estimated prevalence of heterozygous FH (HeFH) of 1 in every 500 [12]. However, a recent study showed that HeFH was present twice as often in a large Danish population (1 per 200–250). In this study, Benn and co-workers applied the Dutch Lipid Network Criteria to quantify the prevalence of FH in the Copenhagen Heart Study, a prospective study comprising over 69,000 Caucasians of Danish descent. In this cohort, 7.76 % were found to meet the “probable or definite FH” criteria using the Dutch Lipid Network criteria [13]. These numbers are very much in line with the numbers that were found in the study of Sjouke et al. who used a genetic approach focusing on a molecular diagnosis [14]. The database of the nationwide molecular diagnostic center was used to identify 49 HoFH patients. The Hardy-Weinberg equilibrium was used to calculate the prevalence, resulting in a prevalence of 1 in 4,180,597 for HoFH and 1 in 319 for HeFH. In another study where large-scale exome sequencing was performed, approximately 1 in 217 of the patients who were free of CVD was found to carry a mutation in *LDLR*, whereas the prevalence of mutations was 1 in 50 in those patients who suffered from a premature CVD event [15•]. This clearly shows that the true prevalence of FH is probably in the order of 1 in every 200 inhabitants, which would translate in a total of approximately 4.5 million patients with FH in Europe and presumably 35 million people globally. It is of note that regional differences in the prevalence of FH has been described, with higher prevalences in certain populations due to founder effect, for example in South-Africa and Quebec [16].

## Consequences and Clinical Hallmarks of Familial Hypercholesterolemia

Due to impaired clearance, LDL-particles accumulate in the arterial wall leading to an inflammatory response. The endothelial tissue becomes damaged and atherosclerotic plaques are formed [17]. Endothelial damage begins at a young age, which was shown by studies by de Jongh and co-workers, who measured endothelial function by means of flow-mediated dilation (FMD) in children who were diagnosed with HeFH and their non-affected brothers and sisters. It was shown that endothelial function was already impaired in these asymptomatic HeFH patients at the age of 9 to 18 years [18]. The extent of atherosclerosis is further enhanced by other risk factors [19]. It is of note that some risk factors, such as elevated levels of the proatherogenic lipoprotein (a) (Lp (a)), are commonly observed in FH patients [20]. Moreover, triglyceride-rich lipoprotein remnants might contribute to increased CVD risk and premature atherosclerosis in FH [21]. Atherosclerotic plaques are predominantly found in the coronaries, peripheral arteries, and aortic valve [2]. Furthermore, cholesterol can accumulate in the skin leading to xanthomas, which are primarily observed in the tendons at the elbows, hands, and Achilles. Cholesterol depositions are also found around the eyes in the form of xanthelasmata or in the cornea, where it can be observed as an arcus lipoides. The presence of xanthoma is pathognomonic for FH and one of the diagnostic criteria for FH [7]. In FH patients, presence of xanthomas are associated with a threefold increased risk of CVD compared to FH patients without xanthomas [22]. Xanthomas are more frequently seen in HoFH, even at birth or during early childhood [11], but can also be seen in HeFH later in life.

## How to Diagnose Familial Hypercholesterolemia

### Clinical Versus Molecular Diagnosis

There are several diagnostic criteria to diagnose FH based on different phenotypical and molecular scoring algorithms, and the prediction based on these criteria sets do not differ to a great deal [23]. The Dutch Lipid Network Criteria is widely accepted and commonly used [24]. These can be used to calculate a score predicting the likelihood of FH, whereas a score higher than five makes the diagnosis probable (Table 1). Other criteria that are used and internationally validated are the Simon Broom system criteria [25], the MEDPED criteria (Make Early Diagnosis to Prevent Early Death) [26], and the Japanese criteria [27]. Secondary causes of hypercholesterolemia such as proteinuria, hypothyroidism, or medication must be excluded [24].

**Table 1:** Dutch Lipid Clinic Network criteria for diagnosis of heterozygous familial hypercholesterolemia

Group 1: family history	Points
• First-degree relative with known premature coronary heart disease (CHD) (<55 years for men; <60 years for women)	1
• First-degree relative with known LDL cholesterol 95th percentile by age and gender for country	1
• First-degree relative with tendon xanthoma and/or corneal arcus OR	2
• Child(ren) <18 years with LDL cholesterol >95th percentile by age and gender for country	2
Group 2: clinical history	
• Subject has premature CHD (<55 years for men; < 60 years for women)	2
• Subject has premature cerebral or peripheral vascular disease (<55 years for men; <60 years for women)	1
Group 3: physical examination	
• Tendon xanthoma	6
• Corneal arcus in a person <45 years	4
Group 4: biochemical results (LDL cholesterol)	
• >8.5 mmol/L (>325 mg/dL)	8
• 6.5–8.4 mmol/L (251–325 mg/dL)	5
• 5.0–6.4 mmol/L (191–250 mg/dL)	3
• 4.0–4.9 mmol/L (155–190 mg/dL)	1
Group 5: molecular genetic testing (DNA analysis)	
• Causative mutation shown in the *LDLR*, *APOB*, or *PCSK9* genes	8

With the algorithm, a numerical score can be calculated which predicts the change that a subject has FH. It is only possible to score once per group. The highest applicable can be chosen. “Definite FH” >8 points, “Probable FH” 6–8 points, “Possible FH” 3–5 points

Finding a pathogenic mutation in the *LDLR*, *APOB*, or *PCSK9* gene is considered to be the gold standard for diagnosing monogenic causes of FH. However, FH cannot be ruled out in the absence of a known mutation being identified. In fact, in a substantial number of cases, no monogenic defect can be identified [28]. Reported mutation detection rates range from 20 to 95 % [29–31]. It is possible that in these patients mutations in hitherto unidentified FH genes are present. In line with this assumption is the recent finding of mutations in *STAP1* in patients with FH [32]. Carriers of mutations in this gene were characterized by an FH-like phenotype. Little is known however about the role of STAP1 in lipid metabolism. The gene does not seem to be expressed in tissue with an established role in LDL-metabolism, and clearly, the unraveling of the effect of STAP1 mutations warrants further studies. Identification of such novel “FH genes” might have a huge impact on our understanding and treatment of dyslipidemia [33]. Alternatively, the FH phenotype might be due to a number of relatively benign variations in a number of genes, a so-called polygenic form of FH. This hypothesis was tested by Talmud, Humphries and coworkers [3], who indeed showed that the number of LDL-C increasing variations was higher in FH patients in whom no monogenic form was identified compared to controls. It should be emphasized, however, that the LDL-C levels in these subjects were in general lower compared to patients in whom a monogenic defect was identified (LDL-C of 5.87 and 7.03 mmol/L, respectively, *p* = 0.002) [3]. It is important to note that mode of inheritance of these SNPs is not dominant, since the SNPs are located over the whole genome [34]. Furthermore, it is unclear whether this is indeed familial, as the former has implications for genetic screening of relatives whereas non-Mendelian inheritance would not.

## Screening for Familial Hypercholesterolemia

Because of the high prevalence of FH, a systemic approach in screening is a legitimate strategy for public health. There are several screenings methods for FH. In the Netherlands, cascade screening has been exploited for over two decades. Upon identification of the index case, family-directed cascade screening s takes place. Index cases can, for example, be identified by opportunistic screening among patients with a (family) history of a CVD event at a young age. Measuring LDL-C levels in such patients should be performed on a routine basis and, while applying any of the clinical criteria metrics, this can be followed by DNA analysis [35]. When a pathogenic mutation is found, cascade screening can take place identifying the same mutation in first-degree relatives. In the Netherlands, over 27,000 individuals have been diagnosed with FH by this method. Multiple studies have shown that cascade screening is the most cost-effective screenings strategy for FH [36, 37]. Although genetic testing has been widely accepted as the gold standard for the diagnosis, measuring LDL-C levels is obligatory since these levels tailor the extent of therapy being prescribed [38]. It is of note that some patients with pathogenic mutations do not show the expected phenotype of elevated LDL-C levels [39], and given the fact that the LDL-C rather than the mutation is the driver of the CVD risk, any preventive therapy measure should be focused on the clinical phenotype, rather than on the presence of a molecular defect [40].

Another type of screening is universal screening, which involves screening of all individuals in a certain category, for example children of a certain age. So far, this only has been introduced in Slovenia for children at the age of 5 [41]. Universal screening in the USA at 9 and 11 years has been proposed, for example when vaccination takes place. Recently, it has been proposed to consider universal screening in patients under 20 years of age and preferable before puberty [35]. Ideally, this screenings strategy would be integrated with cascade screening afterwards, to maximize the detection rate [42].

## Molecular Diagnosis: New Detection Methods

Several molecular testing methods are being used to detect mutations in any of the established FH genes, including dideoxy sequencing, array-based sequencing (in case of a relatively limited number of mutations in the population), or denaturing high performance liquid chromatography (DHPLC) and melting analysis [43]. For detection of large insertions of deletions, multiplex probe amplification (MLPA) is used [43]. The large disadvantage of the Sanger-based sequence methods is that it is relatively time and labor intensive and this problem is largely overcome by next generation sequencing (NGS) where multiple genes can be analyzed at once. NGS can produce billions of nucleotide reads from a sample of one patient and is relatively inexpensive [44]. NGS can either be used to perform whole genome sequencing (WGS), whole exome sequencing, or targeted subgenome analysis [45], and with these techniques, causative mutations have been identified in a number of patients with monogenetic disease [44]. For FH, NGS has been used in a number of laboratories with differences in success rates [46].

## CVD Risk in FH

In the pre-statin era, patients were considered to be at a 100-fold increased risk for coronary heart disease mortality when aged 20–39 [25]. Statins were introduced in the 1990s and numerous studies have shown that the lowering LDL-C levels by statin therapy results in a reduction in cardiovascular mortality and morbidity [47]. The effect of statins on CVD events in FH patients, however, has not been well addressed. It is widely considered that the aforementioned 100-fold increased risk might be an overestimate. Several studies have been published about the CVD risk in FH patients and the risk ratio (RR) associated with FH range from 3 to 16 [13, 15•, 48]. Benn et al. (2010) used The Danish General Population to estimate the risk of coronary artery disease (CAD) in probable or definite FH using the Dutch Lipid Network criteria and found that FH patients, who were not treated with lipid lowering therapy, were at 13-fold risk for CAD compared to non-FH individuals [13]. When using lipid-lowering therapy, compared to non-FH subject, patients with FH were at a 10-fold risk for CAD. In another study by Huijgen et al., patients with a pathogenic *LDLR* mutation had a shorter event-free survival than their relatives who did not carry that mutation (HR 3.64, 95 % CI = 3.24–4.08, *P* < 0.001) [48]. In a recent study by Do and co-workers, exome sequencing was performed in nearly 10,000 genomes of patients with myocardial infarction (MI) at a young age, as well as controls. It was found that carriers of non-synonymous mutations in the gene coding for the LDLR were at 4.2-fold higher increased risk for MI. This risk was even higher in carriers of an *LDLR* null mutation (13-fold difference) [15•] (see Table 2 for an overview).

**Table 2:** Cardiovascular disease in FH

	CVD risk
Simon Broome Register Group (1991) [25]	
• Deaths from coronary disease FH vs. non FH	SMR: 386 (95 % CI 210 to 639)
Benn et al. (2012) [13]	
• CAD in FH vs. non FH (no lipid lowering therapy)	OR: 13.2 (95 % CI 10.0–17.4)
• CAD in FH vs. non FH (with lipid lowering therapy)	OR: 10.3 (95 % CI 7.8–13.8)
Huijgen et al. (2012) [48]	
• Event free survival in carriers of pathogenic *LDLR* mutation vs. no pathogenic *LDLR* mutation	HR: 3.64 (95 % CI = 3.24–4.08, *p* < 0.001)
Do et al. (2015) [15•]	
• MI in carriers of non-synonymous mutations *LDLR* gene vs. no non-synonymous mutations *LDLR* gene	OR 4.2 (*p* = 3 × 10^−11)
• MI in *LDLR* null mutation vs. no null mutation	OR 13.0 (*p* = 9 × 10^−5)

*FH* familial hypercholesterolemia, *CVD* cardiovascular disease, *SMR* standardized mortality ratio, *CI* confidence interval, *CAD* coronary artery disease, *OR* odds ratio, *HR* hazard ratio, *p p* value, *MI* myocardial infarction, *LDLR* LDL receptor

## Current Therapy

In a number of international guidelines, different LDL-C targets for therapy in FH have been published over the last 5 years. The target for LDL-C varies in these guidelines and ranges from a minimal 50 % reduction of plasma LDL-C to a target LDL-c level below 2.5 or 1.8 mmol/L in FH patients without or with sharply increased risk for CVD, respectively. The recently published ESC/EAS guideline on FH recommends an LDL-C target level below 2.5 mmol/L for adults, below 1.8 mmol/L for adults with coronary heart disease (CHD) or diabetes, and below 3.5 mmol/L for children. Targets are the same for HeFH and HoFH, regardless of age [7]. The Canadian Guidelines by the Canadian Society also recommend target LDL-C based on different risk categories. For patients with high risk (i.e., CAD, peripheral vascular disease (PVD), atherosclerosis or diabetes) and moderate risk, LDL-C target is <2 mmol/L; in the low risk category (Framingham risk score <10 %), an LDL-C reduction of >50 % is recommended [49]. However, the Framingham risk score is not reliable in FH. The recently updated ACC/AHA guidelines (USA) recommend a reduction of >50 % plasma LDL-C levels, acknowledging the fact that no evidence is available supporting a pre-defined target LDL-C level [6].

It is of note that statins, albeit their efficacy in lowering LDL-C, are widely underused in FH patients [13]. Moreover, the LDL-C levels recommended by the EAS/ESC are not met in over half of the FH patients using statins, even in patients who are treated with maximal doses [50].

### Statins

Statin therapy is the cornerstone in the treatment of patients with FH. There is a large and robust amount of evidence showing reduction in cardiovascular events by use of statins [24, 47]. The effectiveness of statins is based up on up-regulation of LDLR by inhibiting HMG-CoA reductase, resulting in lower plasma LDL-C [21]. In their study, Versmissen and colleagues specifically addressed the effect of statins in patients with HeFH [51]. In this non-randomized, retrospective study, it was shown that when treated with statins before onset of CHD, there was a risk reduction of 76 %. Moreover, the risk of myocardial infarction in this statin treated group was similar to that in the general population. In the EAS consensus paper of the EAS of Nordestgaard et al., it is advised to start with a maximal potent statin dose in FH patients, if tolerated [7]. In case LDL-C levels are not achieved, adding ezetimibe, a cholesterol absorption inhibitor, is advised [7].

In some HoFH patients, statins may be effective, although the effects on plasma LDL-C levels are known to be relatively modest [52], which is due to the severe deficiency in LDLR function. Therapy of choice for HoFH patients is weekly LDL-C apheresis [53]. If apheresis is not available, lomitapide (oral microsomal triglyceride transfer protein (MTP) inhibitor) or mipomersen (antisense apoB) can be given in addition to statins to further lower LDL-c [35].

### Ezetimibe

Addition of ezetimibe may be necessary to achieve LDL-C targets. Due to reduced absorption of cholesterol in the bowel, there is a compensatory increase in LDLR on hepatocytes and consequently and 20 % reduction in LDL-C [5]. Ezetimibe could also be prescribed as monotherapy for individuals who are not able to tolerate statins, but is preferably given in combination [54]. In the ENHANCE trial, treatment with a combination of ezetimibe and simvastatin did not result in a significant difference in intima-media thickness (cIMT) in comparison to monotherapy with simvastatin [55]. This unexpected result is likely related to the fact that the cIMT values at the time of enrollment were not increased, and as a consequence, a difference over time was less likely to be reached. However, in the recently published IMPROVE-IT trial, cardiovascular outcomes were evaluated in more than 18,000 patients who were hospitalized for an acute coronary syndrome. The results showed that a combination of ezetimibe and statins resulted in additional LDL-C lowering and improved cardiovascular outcomes [56].

### Novel Therapies

As mentioned before, many individuals with FH are not able to achieve sufficient reduction in LDL-C levels [50]. This unmet need has driven the development of novel therapies for further LDL-C lowering.

### PCSK9 Inhibition

PCSK9 is a serine protease secreted by hepatocytes and is involved in the degradation of the LDLR [10]. Monoclonal antibodies against PCSK9 have been developed and result in increased expression of the LDLR and therefore lowering of LDL-C levels. Following phase 2 studies, PCSK9 inhibitors alirocumab [57••] and evolocumab [58••] were compared to placebo in subjects with heterozygous FH. Use of alirocumab every 2 weeks resulted in LDL-C reduction of 67 % and subcutaneous injections of evolocumab every 4 weeks resulted in a 70 % lowering of LDL-C. Recently, the results of the OSLER trial showed that evolocumab was efficacious and safe [59].

### CETP Inhibition

Cholesterylester transfer protein (CETP) is a protein that facilitates exchange of cholesteryl esters for triglycerides between and among HDL particles and apoB-containing lipoproteins (VLDL, intermediate-density protein (IDL), and LDL particles) [60]. Blocking this transport by inhibiting CETP results in an increase in HDL-C and apolipoprotein A1 and a decrease in atherogenic lipoproteins such as LDL-C and Lp (a) [61]. The recently published results of the REALIZE trial showed that treatment with the CETP inhibitor anacetrapib for 1 year resulted in substantial reductions in LDL-C concentration [62]. The mechanism by which CETP reduction gives a reduction in LDL-C is unknown. Rader and co-workers recently showed that CETP inhibition by anacetrapib reduces LDL-apoB-100 levels by increasing the rate of apoB-100 fractional clearance and increasing affinity for the LDLR [63]. Whether CETP inhibition leads to a reduction in cardiovascular risks still has to be established in an outcome study.

### Novel Therapies for the Treatment of Homozygous FH

Mipomersen is an antisense oligonucleotide that binds to apoB messenger RNA which results in a decreased apoB synthesis [64]. It is approved for treatment in patients with HoFH in the USA but not in Europe. It has shown to lower plasma LDL-C in 21 % in patients with HoFH [65] and 28 % in patients with HeFH [66].

Lomitapide inhibits MTP at the hepatocytes and blocks the transfer of triglycerides into VLDL in the liver and chylomicrons in the bowel [65]. Lomitapide is approved for treatment of HoFH. LDL-C reductions of 50, 44, and 38 %, respectively, at 26, 56, and 78 weeks have been described [67].

## Conclusion

FH is a common inherited disease which leads to premature CVD and atherosclerosis, and early treatment is needed. FH is widely underdiagnosed and LDL-C targets are often not achieved so new therapies are being developed to overcome this problem. This review gives an up to date overview of clinically relevant information on FH.
